# Exploring the causal effects of the gut microbiome on serum lipid levels: A two-sample Mendelian randomization analysis

**DOI:** 10.3389/fmicb.2023.1113334

**Published:** 2023-02-16

**Authors:** Gongjie Guo, Yonglin Wu, Yingjian Liu, Zixian Wang, Guifeng Xu, Xipei Wang, Feiqing Liang, Weihua Lai, Xiao Xiao, Qian Zhu, Shilong Zhong

**Affiliations:** ^1^School of Medicine, South China University of Technology, Guangzhou, China; ^2^Department of Pharmacy, Guangdong Provincial People's Hospital, Guangdong Academy of Medical Sciences, Southern Medical University, Guangzhou, Guangdong, China; ^3^Guangdong Provincial Key Laboratory of Coronary Heart Disease Prevention, Guangdong Cardiovascular Institute, Guangdong Provincial People’s Hospital, Guangdong Academy of Medical Sciences, Southern Medical University, Guangzhou, Guangdong, China; ^4^School of Pharmaceutical Sciences, Southern Medical University, Guangzhou, China; ^5^Laboratory of Phase I Clinical Trials, Guangdong Provincial People’s Hospital, Guangdong Academy of Medical Sciences, Southern Medical University, Guangzhou, Guangdong, China; ^6^Research Center of Medical Sciences, Guangdong Provincial People’s Hospital, Guangdong Academy of Medical Sciences, Southern Medical University, Guangzhou, Guangdong, China; ^7^School of Pharmaceutical Sciences, Sun Yat-sen University, Guangzhou, China

**Keywords:** gut microbiota, dyslipidemia, serum lipid levels, Mendelian randomization, causal relationship

## Abstract

**Background:**

The gut microbiome was reported to be associated with dyslipidemia in previous observational studies. However, whether the composition of the gut microbiome has a causal effect on serum lipid levels remains unclear.

**Objective:**

A two-sample Mendelian randomization (MR) analysis was conducted to investigate the potential causal relationships between gut microbial taxa and serum lipid levels, including low-density lipoprotein cholesterol (LDL-C), high-density lipoprotein cholesterol (HDL-C), total cholesterol (TC), and log-transformed triglyceride (TG) levels.

**Materials and methods:**

Summary statistics of genome-wide association studies (GWASs) for the gut microbiome and four blood lipid traits were obtained from public datasets. Five recognized MR methods were applied to assess the causal estimates, among which, the inverse-variance weighted (IVW) regression was used as the primary MR method. A series of sensitivity analyses were performed to test the robustness of the causal estimates.

**Results:**

The combined results from the five MR methods and sensitivity analysis showed 59 suggestive causal associations and four significant causal associations. In particular, genus *Terrisporobacter* was associated with higher LDL-C (*P*_IVW_ = 3.01 × 10^−6^) and TC levels (*P*_IVW_ = 2.11 × 10^−4^), phylum *Actinobacteria* was correlated with higher LDL-C level (*P*_IVW_ = 4.10 × 10^−4^), and genus *Oscillospira* was associated with lower TG level (*P*_IVW_ = 2.19 × 10^−6^).

**Conclusion:**

This research may provide novel insights into the causal relationships of the gut microbiome on serum lipid levels and new therapeutic or prevention strategies for dyslipidemia.

## 1. Introduction

Dyslipidemia is a common lifestyle-related disease and one of the crucial risk factors for the development of cardiovascular disease (CVD), which is a major health issue and the leading cause of mortality worldwide. As one of the defects in lipid metabolism, dyslipidemia is characterized by abnormal blood lipid levels, which may include a combination of increased low-density lipoprotein cholesterol (LDL-C), total cholesterol (TC), and triglyceride (TG) or decreased high-density lipoprotein cholesterol (HDL-C) (Kopin and Lowenstein, 2017). Aggressive dyslipidemia diagnosis and treatment can help lower the morbidity and mortality rates of CVD (The Human Microbiome Project Consortium, 2012).

The gut microbiota refers to the whole microbial population that colonizes the intestinal tract, including bacteria, archaea, viruses, and protozoans. Most of them are within the large intestine, where more than 70% of all microbes in the body have been discovered (Piccioni et al., 2021). In recent years, the relationship between gut microbiota and disease pathogenesis has attracted much attention from the academe. The composition of the gut microbiota varies substantially with age, sex, lifestyle, or environmental factors (De Filippo et al., 2010; Jin et al., 2017; Takagi et al., 2019). The gut microbiota participates in regulating lipid metabolism in the host leading to a change in blood lipid levels (Visconti et al., 2019), which indicates that it may be a potential risk factor for dyslipidemia. Previous studies have confirmed the link between gut microbiota and dyslipidemia. A cohort study by Fu et al. (2015) pointed out the important role of the gut microbiota in the variations in TG and HDL-C levels. Animal studies have also reported that host lipid metabolism can be affected by the gut microbiota through multiple direct and indirect mechanisms (Ghazalpour et al., 2016; Schoeler and Caesar, 2019). In addition, Miyajima et al. (2022) found that several bacterial taxa, including *Prevotella 9* and *Bacteroides* in men and *Akkermansia* and *Escherichia*/*Shigella* in women, are associated with serum lipid profiles in a Japanese cohort. Although the correlations between gut microbiota and dyslipidemia have been described by previous studies, the causal relationship between them remains unclear, and interpreting the causation would be much more complex and challenging.

A great number of genome-wide association studies (GWASs) have analyzed the correlations between genetic variation and diseases or phenotypes. Mendelian randomization (MR) is a powerful statistical method to evaluate causation. It uses genetic variations remarkably associated with exposure as instrumental variables (IVs) to assess the causal relationship of the exposure to the outcome (Burgess et al., 2015). Two-sample MR analysis can measure causal estimates based on single-nucleotide polymorphism (SNP)-exposure and SNP-outcome associations extracted from independent GWAS studies. Recent MR studies have explored the causal relationships between different exposures to dyslipidemia. However, to our knowledge, no MR analysis has been performed to evaluate the causal association of the gut microbiota with serum lipid levels.

Therefore, we obtained summary statistics from public large-scale GWAS consortiums and conducted a two-sample MR analysis to assess the causal relationships between the composition of gut microbiota and serum lipid levels to identify potentially modifiable risk factors for dyslipidemia.

## 2. Materials and methods

**Figure 1:**
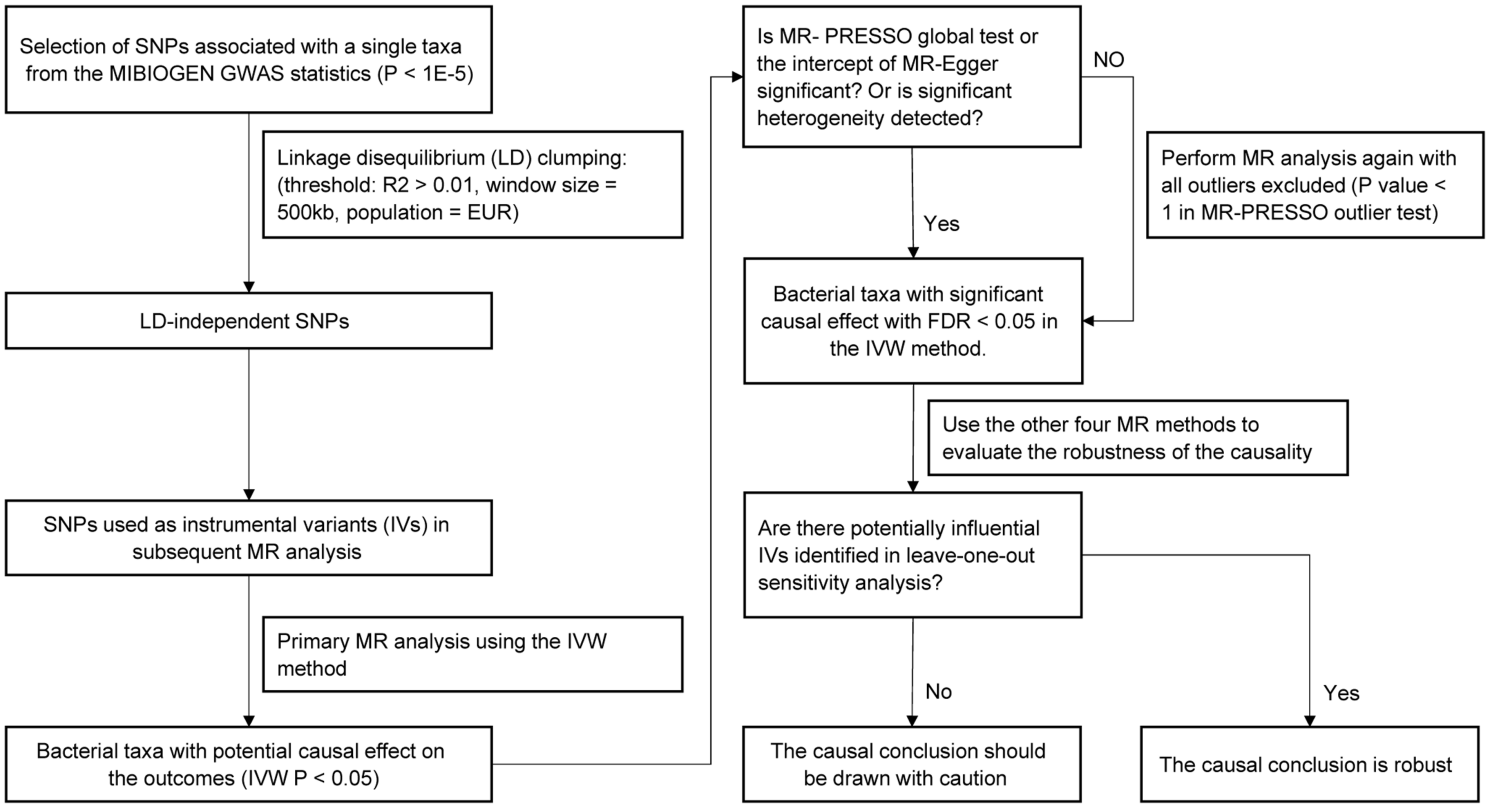
Flow chart of the MR analysis.

The study flow is illustrated in Figure 1. Two-sample MR method was applied to assess the causal relationship between gut microbiota composition and serum lipid levels. The SNPs used as IVs in summary-level MR analysis should satisfy the following three key assumptions of MR analysis (Davey Smith and Hemani, 2014): (1) the relevance assumption: IVs are strongly correlated with the exposure of interest; (2) the independence assumption: IVs are not associated with confounders related to the exposure or outcome; (3) the exclusion assumption: IVs only affect the outcome through the exposure. Two-sample MR analysis should be performed under these assumptions to prevent the causal estimates from being biased.

### 2.1. Data sources

Genetic predictors for human gut microbiota composition were obtained from the summary statistics of a large-scale, multi-ethnic, microbiota-based GWAS meta-analysis (the MiBioGen study), which consists of 18,340 participants from 24 cohorts (Kurilshikov et al., 2021). In the present study, the microbial composition was profiled by targeting three distinct variable regions of the 16S RNA gene, and all datasets were rarefied to 10,000 reads per sample. The direct taxonomic binning method was applied in taxonomic classification. A total of 211 bacterial taxa presented in more than 10% of the samples in each cohort were included in the analysis of the relationship between the host and gut microbiota composition. Fifteen unknown bacterial taxa were excluded, and 196 taxa (119 genera, 32 families, 20 orders, 16 classes, and 9 phyla) were included in the MR analysis as the exposures. In addition, the GWAS summary statistics of another two large-scale metagenomics mbQTL studies, as listed in Table 1, were included as the candidate replication datasets to test the reliability of significant causal associations.

**Table 1:** Summary information of the datasets employed in the current MR study.

Traits	*N*	nSNP	Data type	Population	References
Gut microbiota (discovery)	18,340	NA	Continuous	72.3%European	Kurilshikov et al. (2021)
Gut microbiota (replication)	5,959	7,980,478	Continuous	European	Qin (2022)
	7,783	5,584,686	Continuous	European	Lopera-Maya et al. (2022)
Serum lipids	1,320,016	47 M	Continuous	European	Graham et al. (2022)

*N*, the total sample size; nSNP, number of the SNP used as the IVs for the MR analyses.

The summary statistics for serum lipid levels (LDL-C, HDL-C, TC, and log-transformed TG) were assessed from the Global Lipids Genetics Consortium–aggregated GWAS meta-analysis of 201 primary studies, involving 1,654,960 participants from five genetic ancestry groups. Ancestry-specific GWAS summary statistics for the four lipid traits in a European cohort were used in our MR analysis (Graham et al., 2022). No ethical approval was needed, because all data used in the current study were obtained from publicly available GWAS datasets.

### 2.2. Selection of instrumental variables

We performed strict quality control (QC) on the SNPs in the microbiota-based GWAS summary study to select valid IVs for MR analysis. We used a relatively loose *p* value of 1 × 10^−5^ to select SNPs associated with each bacterial taxon as candidate IVs. Afterward, independent IVs were selected for each bacterial taxon by performing linkage disequilibrium (LD) analysis to prevent the causal estimates from being biased. We clumped each set of microbiota-associated SNPs to obtain independent instruments (*R*^2^ < 0.01, window size = 500 kb) with the genome samples from the 1,000 Human Genomes Project (EUR) (The 1000 genomes project consortium, 2012) used as the LD reference panel. Among the SNPs in substantial LD, whose *R*^2^ was greater than the threshold, only the SNPs with the strongest association with the corresponding bacterial taxa could remain as the candidate IVs. The relevance assumptions of MR analysis require that the selected IVs should be substantially associated with the exposure. *F*-statistics (Burgess et al., 2011) is a method used to measure the strength of the IVs in MR analysis. Therefore, the total instrument F-statistic for each bacterial taxon was calculated by the following formula to evaluate the strength of the association between the candidate IVs and the corresponding taxon:


$$F=\frac{PVE\left(n-k-1\right)}{\left(1-PVE\right)}$$

where PVE represents the proportion of exposure variation by the selected IVs, *n* represents the total exposure GWAS population size, and *k* represents the total number of IVs. The PVE of each IV was calculated using the formula:


$$PVE=\frac{2*\beta^{2}*MAF*\left(1-MAF\right)}{2*\beta^{2}*MAF*\left(1-MAF\right)+2*Se^{2}*n*MAF*\left(1-MAF\right)}$$

where MAF is the minor allele frequency, *β* is the beta value, and Se is the standard error (Shim et al., 2015). Through fixing *k* equals 1, per-SNP F-statistics were also calculated using the first formula, and the weak IVs with *F*-statistics less than 10 were excluded from the candidate IV sets.

In addition, the existence of pleiotropic variants related to other unknown confounding factors may violate the independent assumptions of the MR analysis, which could probably result in the bias of causal estimates. Therefore, we applied the MR–Egger and MR–PRESSO approaches to test the horizontal pleiotropy of all candidate IVs that passed the QC steps (Verbanck et al., 2018). In the MR–Egger method, we considered that the horizontal pleiotropy of the IVs was not remarkable enough to cause an impact on the causal conclusions if the absolute value of the intercept was <0.1 and the corresponding *p-*value was >0.05. Similarly, we drew the same conclusion that no significant pleiotropy exists if the MR–PRESSO global test gave a *p* value of >0.05. Once horizontal pleiotropy was detected, we took a step further to conduct the MR–PRESSO outlier test and removed the potentially outlying SNPs.

### 2.3. MR estimates

In the current study, we performed a two-sample MR analysis to assess the causal relationship between gut microbiota composition and serum lipid levels. For the bacterial taxa with at least three IVs, we applied multiple different robust MR methods, including inverse-variance weighted (IVW) model (Burgess et al., 2013), maximum likelihood estimator (MLE) (Pierce and Burgess, 2013), MR–Egger regression (Bowden et al., 2015), weighted median estimator (WLE) (Bowden et al., 2016), and weighted mode-based estimator (Hartwig et al., 2017), to investigate their causal links to the outcomes. If the causal estimates of more than three MR methods were nominally significant, gut microbiota taxa could be considered to have potential causal effects on blood lipids.

Among the five MR methods, we used the IVW method as the primary method to identify potential causation, as it is acknowledged as the most powerful statistic method of summary-level MR. It combines the Wald ratios of each valid IV to assess the total effect of exposure variables on the outcome through a meta-analysis approach (Burgess et al., 2013). The IVW regression model can provide an unbiased estimate of the causal effect robustly when no SNPs violate the IV assumption of independence (inexistence of horizontal pleiotropy). When performing the IVW approach, fixed-effects or random-effects regression models are available for analysis. If excess existence of heterogeneity (*p* < 0.05) is detected in the IVs, a random-effects IVW model is more recommended to be used rather than a fixed-effects IVW model.

The MR–Egger method is exploited to the causal estimates based on the slope of a weighted linear regression of the IV–outcome associations on the IV–exposure associations with the Instrument Strength Independence of Direct Effect (InSIDE) assumption satisfied (Bowden et al., 2015). Different from the IVW method, where the intercept of IVW forced linear regression is zero, we can evaluate the average pleiotropic effect according to the intercept of the MR–Egger regression. In particular, the InSIDE assumption allows the genetic instruments to have pleiotropic effects independent of the associations between the variants and exposure, which weakens the IV assumption of independence. That is to say, the causal estimates from the MR–Egger method would still be robust to horizontal pleiotropy under this assumption. However, the MR–Egger model may give an inaccurate causal effect estimate when influential data points or outliers are present in the IVs, which results in a lowered power for the MR–Egger model to draw a valid causal inference than the IVW method.

Median-based estimator and mode-based estimator further weaken the IV assumptions of MR analysis. The median-based estimator can provide unbiased causal estimates despite the existence of unbalanced horizontal pleiotropy, even when the ratio of invalid SNPs is up to 50%. In the mode-based estimator approach, SNPs are divided into different subclasses, in which the IVs exhibit similar causal effects. If all IVs in the largest subclasses are valid, the mode-based estimator can also give consistent and unbiased causal estimates.

### 2.4. Sensitivity analysis

In summary-level MR analysis, the presence of IVs with substantial heterogeneity may bias the causal estimates, lessening the reliability of the causal conclusions. Even when all SNPs are valid instruments and satisfy the three MR assumptions, some heterogeneity effects may still exist in the IVs. Therefore, variant-specific IVW regression was utilized to test the heterogeneity of each IV. If the *p-*value of Cochran’s *Q* statistic in the heterogeneity test is lower than 0.05, we would have enough evidence to consider the presence of a considerable heterogeneity among the IVs (Bowden et al., 2018).

Leave-one-out sensitivity analysis was also applied to detect possibly influential SNPs and test the robustness of the causal conclusions (Burgess, 2014). In this step, the causal estimates were re-assessed with each SNP removed from the IVs in turns. If certain SNPs had substantial correlations with the exposure, then these SNP were likely to dominate the causal estimate. Therefore, we should assess the robustness again by re-estimating the causal effect with all such SNPs excluded.

### 2.5. Bidirectional MR analysis

An additional reverse MR analysis was conducted to explore the reverse causal relationship of the blood lipid levels (as exposures) to the three identified bacterial taxa (as outcomes). The procedures of the reverse MR analysis were the same as the abovementioned MR analysis.

### 2.6. Tools

Data cleaning was conducted using Python and Jupyter Notebook. All MR analyses were conducted using R. The five robust MR methods and the sensitivity analysis were performed using the R package “TwoSampleMR” (version 0.5.6), and MR–PRESSO analysis was performed by the R package “MRPRESSO” (version 1.0).

## 3. Results

### 3.1. Selection of instrumental variables

We selected 653, 1,300, 1,640, 2,566, and 7,834 SNPs associated with gut microbiota in the phylum, class, order, family, and genus levels, respectively, at a relatively loose significance level (*p* < 1 × 10^−5^). After LD clumping and harmonization, the number of candidate IVs associated with a certain bacterial taxon for each outcome varied from 3 to 22. No evidence of horizontal pleiotropy effects was detected in the MR − PRESSO global test (*p* > 0.05), which was also shown consistently by the intercept of the MR − Egger regression. In all analyses for serum lipid levels, all the *F*-statistics of the IVs were >10, indicating no evidence of weak instrument bias.

### 3.2. MR analysis

The causal relationship between each pair of bacterial taxa and serum lipid levels was tested by five MR methods. In the primary analysis, we applied the IVW method to assess the causal relationship between gut microbiota and serum lipid levels, and then identified 63 suggestive associations (*P*_IVW_ < 0.05) for 53 bacterial taxa. Among the 63 taxa, 21, 15, 16, and 16 taxa were related to HDL-C, LDL-C, TC, and TG, respectively (Supplementary Tables S2–S5). After multiple-testing correction, four significant causal associations (false discover rate [FDR] < 0.05) were detected: genus *Terrisporobacter* on LDL-C (Estimate = 0.0348, 95% confidence interval [CI]: 0.0202–0.0494, *FDR* = 5.90 × 10^−4^), phylum Actinobacteria on LDL-C (Estimate = 0.0315, 95% CI: 0.0140–0.0490, FDR = 0.040), genus *Terrisporobacter* on TC (Estimate = 0.0318, 95% CI: 0.0150–0.0486, FDR = 0.041), and genus *Oscillospira* on TG (Estimate = −0.0271, 95% CI: −0.0415 to −0.0127, FDR = 0.043). In addition, four taxa had promising causal relationships with TG (0.05 < FDR < 1). In addition, 16 taxa, which include all four identified taxa, showed suggestive causal associations with at least two outcome traits.

**Figure 2:**
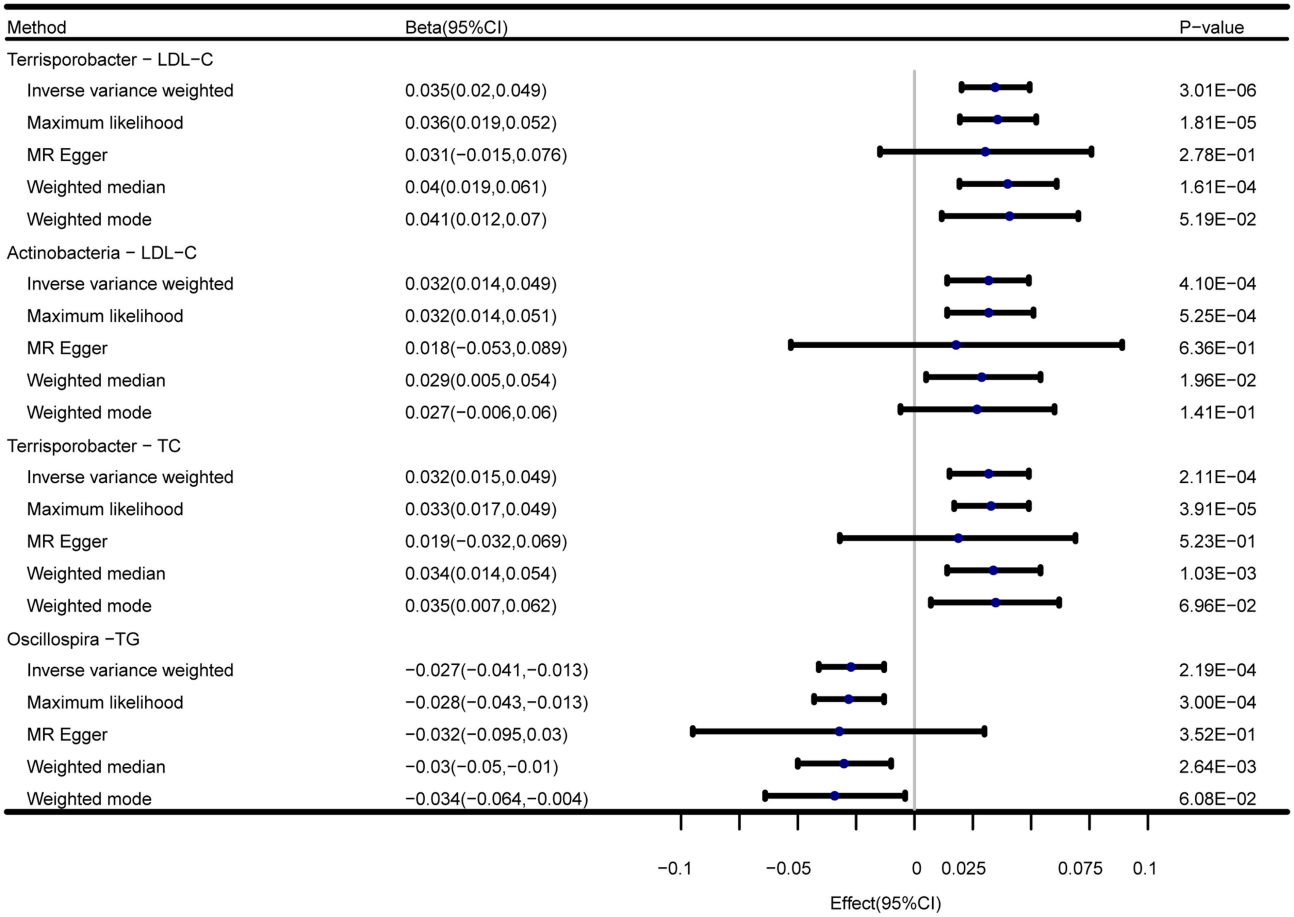
Forest plots of the MR results of the four identified causal associations.

**Figure 3:**
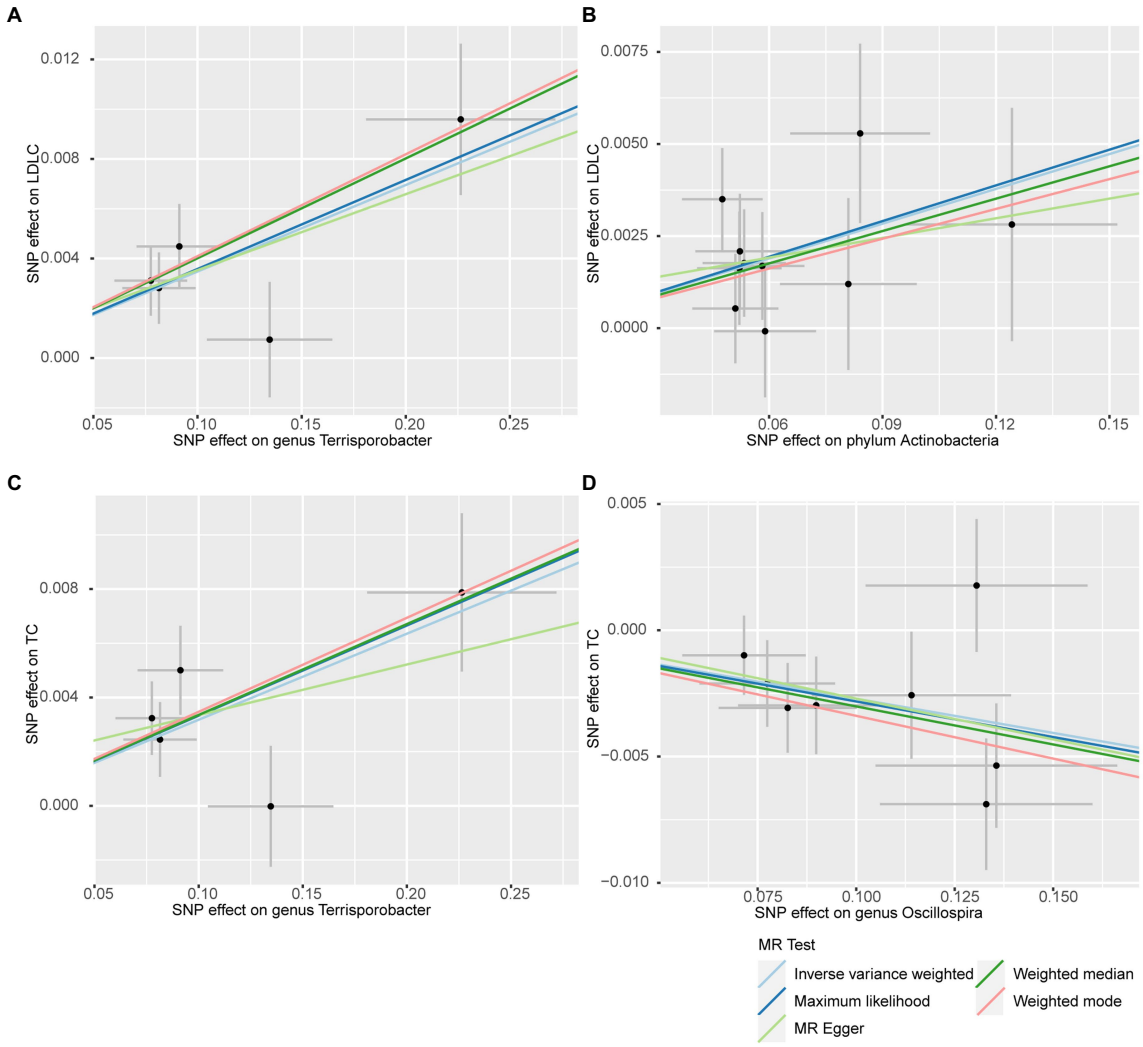
Scatter plots for the causal effects of the identified bacterial taxa on serum lipids. **(A)** *Terrisporobacter* (genus)–LDL-C. **(B)** Actinobacteria (phylum)–LDL-C. **(C)** *Terrisporobacter* (genus)–TC. **(D)** *Oscillospira* (genus)–TG. (Light blue line: inverse-variance weighted; blue line: maximum likelihood; light green line: MR Egger; green line: weighted median estimator; red line: weighted model-based estimator).

In addition to the IVW method, four robust MR methods, namely, MR–Egger, maximum likelihood, weighted mode-based estimator, and weighted median-based estimator, were applied to evaluate the reliability of the causal estimates in our analyses. The full results of all MR methods for the 63 suggestive associations identified in the primary analysis are shown in Supplementary Table S6. For the four significant associations detected by the IVW method, at least three MR methods generated similar and significant causal estimates (Table 1; Figure 2), namely, genus *Terrisporobacter* on LDL-C (*P*_IVW_ = 3.01 × 10^−6^, *P*_Maximum likelihood_ = 1.81 × 10^−5^, *P*_weighted median_ = 1.61 × 10^−4^), phylum Actinobacteria on LDL-C (*P*_IVW_ = 4.10 × 10^−4^, *P*_Maximum likelihood_ = 5.25 × 10^−4^, *P*_weighted median_ = 1.96 × 10^−2^), genus *Terrisporobacter* on TC (*P*_IVW_ = 4.10 × 10^−4^, *P*_Maximum likelihood_ = 5.25 × 10^−4^, *P*_weighted median_ = 1.96 × 10^−2^), and genus *Oscillospira* on TG (*P*_IVW_ = 4.10 × 10^−4^, *P*_Maximum likelihood_ = 5.25 × 10^−4^, *P*_weighted median_ = 1.96 × 10^−2^). Clearly, these significant results are highly consistent with those obtained using IVW regression, indicating the robustness of the identified association signals. The scatter plots of the SNP effect sizes for the four significant associations are shown in Figure 3.

The results of the reverse MR analysis are shown in Supplementary Table S7. However, no significant causal estimates were detected by the five MR methods, which indicated no evidence of causal effect from blood lipid levels to identified bacterial taxa.

### 3.3. Sensitivity analysis

**Figure 4:**
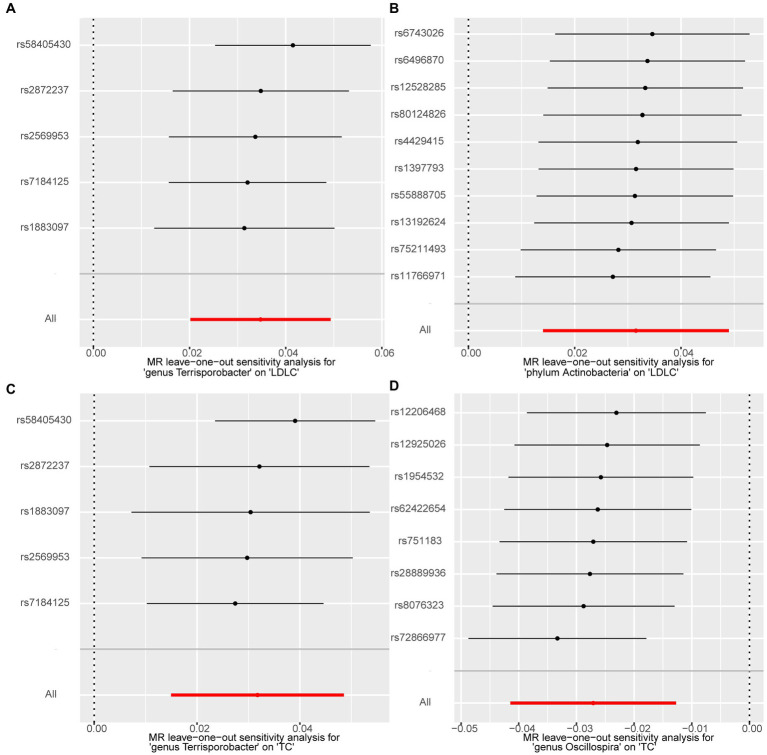
Forest plots of leave-one-out sensitivity analysis for the four identified associations. **(A)** *Terrisporobacter* (genus)–LDL-C. **(B)** Actinobacteria (phylum)–LDL-C. **(C)** *Terrisporobacter* (genus)–TC. **(D)** *Oscillospira* (genus)–TG.

We performed a series of sensitivity analysis to test the heterogeneity and horizontal pleiotropy of the selected IVs to evaluate the robustness of the causal estimates for all the 63 suggestive associations identified by IVW. No heterogeneity effect was detected in the heterogeneity test (*p* > 0.05). The intercept of the MR–Egger regression did not remarkably deviate from zero, and the *p-*value of the MR–PRESSO global test was above 0.05, indicating the absence of horizontal pleiotropy and that no IVs behaved as potential outliers (Table 2). A leave-one-out sensitivity analysis was used to confirm the reliability and stability of the causal effects of the four identified associations. All SNPs of the four identified causal relationships displayed no sensitivity to the results, indicating strong causal links from the identified taxa to the corresponding outcome traits (Figure 4).

**Table 2:** Five MR models’ estimation of the causal relationships between identified bacterial taxa and serum lipid levels and tests for heterogeneity and horizontal pleiotropy.

Exposure	Outcome	Method	nSNP	Beta (95%CI)	*p*-value	*F*-statistics	*P*_Heterogeneity_	*P*_Pleiotropy_	*P*_Global Test_
genus.Terrisporobacter.id.11348	LDL-C	Inverse-variance weighted	5	0.035 (0.02, 0.049)	3.01E-06	21.25	0.28	0.86	0.61
		Maximum likelihood		0.036 (0.019, 0.052)	1.81E-05				
		MR Egger		0.031 (−0.015, 0.076)	2.78E-01				
		Weighted median		0.04 (0.019, 0.061)	1.61E-04				
		Weighted mode		0.041 (0.012, 0.07)	5.19E-02				
phylum.Actinobacteria.id.400	LDL-C	Inverse-variance weighted	10	0.032 (0.014, 0.049)	4.10E-04	22.11	0.79	0.71	0.51
		Maximum likelihood		0.032 (0.014, 0.051)	5.25E-04				
		MR Egger		0.018 (−0.053, 0.089)	6.36E-01				
		Weighted median		0.029 (0.005, 0.054)	1.96E-02				
		Weighted mode		0.027 (−0.006, 0.06)	1.41E-01				
genus.Terrisporobacter.id.11348	TC	Inverse-variance weighted	5	0.032 (0.015, 0.049)	2.11E-04	21.25	0.22	0.63	0.46
		Maximum likelihood		0.033 (0.017, 0.049)	3.91E-05				
		MR Egger		0.019 (−0.032, 0.069)	5.23E-01				
		Weighted median		0.034 (0.014, 0.054)	1.03E-03				
		Weighted mode		0.035 (0.007, 0.062)	6.96E-02				
genus.Oscillospira.id.2064	TG	Inverse-variance weighted	8	−0.027(−0.041, −0.013)	2.19E-04	21.74	0.45	0.87	0.56
		Maximum likelihood		−0.028(−0.043, −0.013)	3.00E-04				
		MR Egger		−0.032(−0.095, 0.03)	3.52E-01				
		Weighted median		−0.03(−0.05, −0.01)	2.64E-03				
		Weighted mode		−0.034(−0.064, −0.004)	6.08E-02				

nSNP, number of the SNP used as the IVs for the MR analyses; 95% CI, 95% confidence interval; *P*_Heterogeneity_, *p-*value of the heterogeneity test; *P*_Pleiotropy_, *p*-value of the intercept of the MR Egger; *P*_Global test,_ *p*-value of the MR-PRESSO global test.

### 3.4. Replication analysis

**Figure 5:**
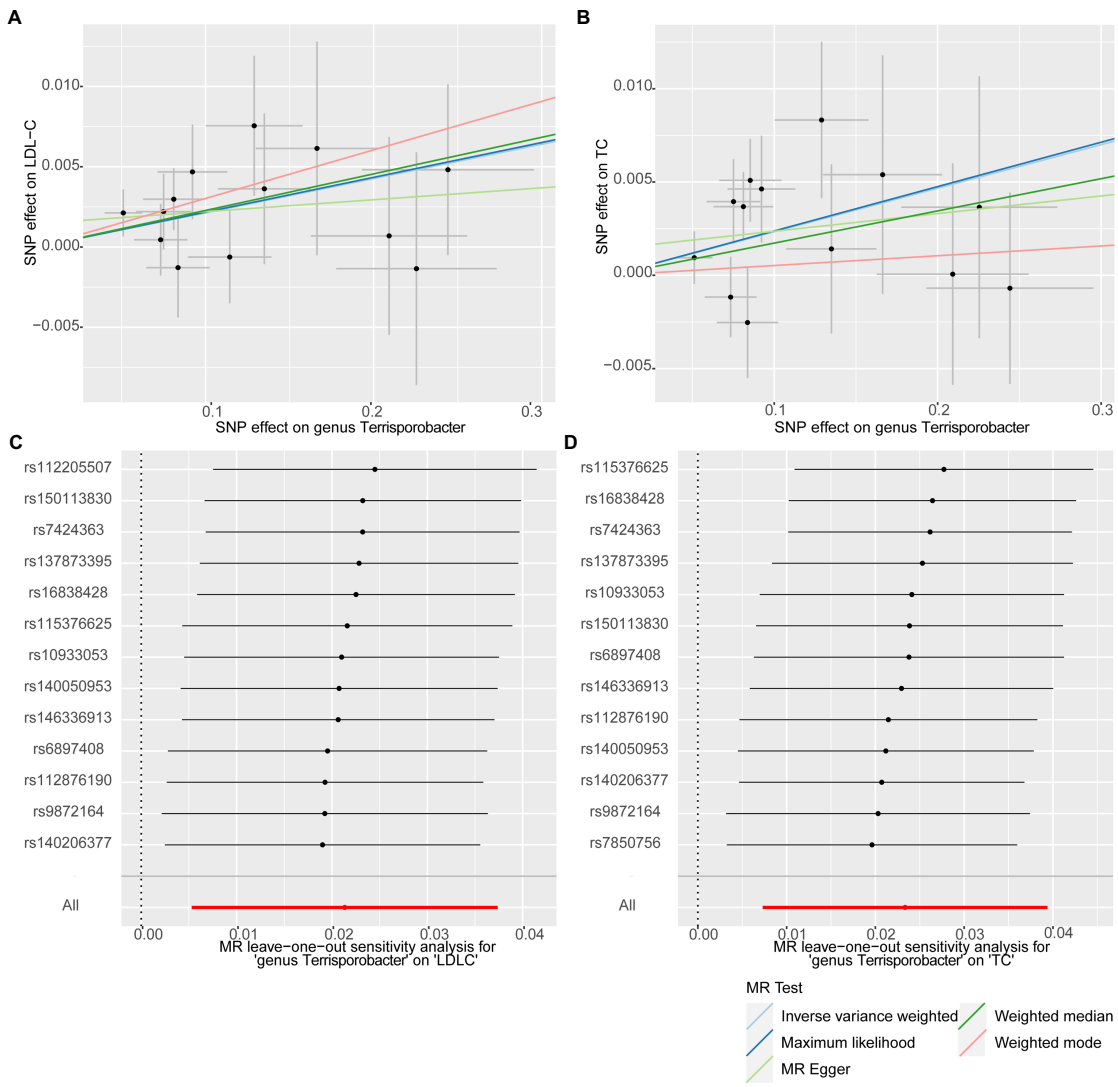
Plots of the significant results of the replication MR analysis. Scatter plots for the causal effects: **(A)** *Terrisporobacter* (genus)–LDL-C. **(B)** *Terrisporobacter* (genus)–TC. Forest plots of leave-one-out sensitivity analysis: **(C)** *Terrisporobacter* (genus)–LDL-C. **(D)** *Oscillospira* (genus)–TC.

In this stage, we searched these two genera *Terrisporobacter* and *Oscillospira* in the candidate replication datasets. However, only the GWAS summary statistics for the genus *Terrisporobacter* as well as the species *Terrisporobacter othiniensis*, which were reported in the study conducted by Qin (2022), could be obtained. An additional replication MR analysis was conducted to test the robustness of the causality from the genus *Terrisporobacter* to the blood lipid levels. The causal effects of genus *Terrisporobacter* on LDL-C (*P*_IVW_ = 9.13 × 10^−3^, *P*_Maximum likelihood_ = 9.74 × 10^−3^, *P*_weighted median_ = 4.13 × 10^−2^) and TC (*P*_IVW_ = 4.33 × 10^−3^, *P*_Maximum likelihood_ = 3.95 × 10^−3^) were successfully replicated, as shown in Table 3. However, no evidence of causal effects of the genetically predicted *Terrisporobacter othiniensis* on serum lipid levels *w*as found. The plots for the significant results of the replication MR analysis are shown in Figure 5. The full results of the replication analysis are listed in Supplementary Table S8.

**Table 3:** Replication of the causalities from *Terrisporobacter* to LDL-C and TC.

Exposure	Outcome	Method	nSNP	Beta (95%CI)	*P*-value	*F*-statistics	*P*_Heterogeneity_	*P*_Pleiotropy_	*P*_Global Test_
Genus Terrisporobacter	LDL-C	Inverse-variance weighted	13	0.021 (0.005,0.037)	9.13E-03	0.88	0.44	21.98	0.90
		Maximum likelihood		0.022 (0.005,0.038)	9.74E-03				
		MR Egger		0.007(−0.03,0.045)	7.06E-01				
		Weighted median		0.023 (0.001,0.045)	4.13E-02				
		Weighted mode		0.03(−0.005,0.065)	1.16E-01				
Genus Terrisporobacter	TC	Inverse-variance weighted	13	0.023 (0.007,0.039)	4.33E-03	0.39	0.45	21.87	0.42
		Maximum likelihood		0.024 (0.008,0.04)	3.95E-03				
		MR Egger		0.009(−0.029,0.048)	6.40E-01				
		Weighted median		0.017(−0.005,0.039)	1.21E-01				
		Weighted mode		0.005(−0.036,0.046)	8.06E-01				

nSNP, number of the SNP used as the IVs for the MR analyses; 95% CI, 95% confidence interval; *P*_Heterogeneity_, *p-*value of the heterogeneity test; *P*_Pleiotropy,_ *p-*value of the intercept of the MR Egger; *P*_Global test,_ *p*-value of the MR-PRESSO global test.

## 4. Discussion

In this study, we used a two-sample MR method to explore the directional causal relationship between gut microbiota and blood lipids based on large-scale GWAS summary statistics. After conducting a series of QC procedures, the instrumental variables were excluded that might bias the causal estimates. Besides, the results of the sensitivity analysis detected no heterogeneity, horizontal pleiotropy, or potentially influential IV, suggesting that the causal inferences of our MR study were robust enough. Our research is the first MR study focusing on the causal links between gut microbiota and serum lipid levels.

In total, we detected 63 promising associations between bacterial taxa with blood lipid levels. After the correction of multiple testing, we found that the genus *Terrisporobacter* was causally associated with LDL-C and TC, phylum Actinobacteria was causally associated with LDL-C, and genus *Oscillospira* was causally related to TG. No reverse causal relationship was detected in bidirectional MR analyses.

A compelling finding of this study is that the genus *Terrisporobacter* showed substantial positive causal effects on LDL-C and TC, which was also confirmed by the replication MR analysis. Although very little is known about *Terrisporobacter*, our finding is similar to the results of Lee et al. (2020), who discovered that the abundance of *Terrisporobacter* is positively correlated with CRP, TG, BMI, and weight and negatively correlated with HDL-C after the consumption of symbiotic beverages. In addition, the genus *Terrisporobacter* has been linked to short-chain fatty acids (SCFAs) and oxidative stress in an animal study (Li et al., 2021). A randomized controlled trial in children with type 1 diabetes identified a remarkably higher relative abundance of *Terrisporobacter* in the placebo group compared with the prebiotic group at 3 months (Ho et al., 2019). All these studies suggest that *Terrisporobacter* is a pathogenic bacterium. Xiao et al. (2020) reported that 8 bacterial genera including *Terrisporobacter* were significantly and positively correlated to TC, TG, LDL-C, and negatively related to HDL-C. Their study also demonstrated that decreased abundance of *Terrisporobacter* might increase the levels of phospholipids, purine, carboxylic acids, unsaturated fatty acids, and bile acids. Some of the abovementioned metabolites, for instance, phospholipids (Henderson et al., 2019) and bile acids (Li and Chiang, 2015), have been explicitly shown to be associated with reduced cholesterol levels. Thus, we presumed that the genus *Terrisporobacter* might involve in the regulation of enzymes in bile acid metabolism or lipid biosynthesis, eventually leading to higher serum lipid levels and dyslipidemia. However, to date, studies on the role of *Terrisporobacter* on serum lipid levels remain limited owing to the lack of research on *Terrisporobacter*. Therefore, more studies on genus *Terrisporobacter* are needed to demonstrate the underlying mechanisms and causal effects of these identified associations in the future.

Our MR analysis also identified the substantial and positive causal relationship of phylum *Actinobacteria* on TC. As one of the basic phyla of gut microbiota, *Actinobacteria* is a controversial intestinal flora. Some previous studies reported that *Actinobacteria* is beneficial for human health (Cani et al., 2007) while some suggested the opposite. Nonetheless, growing evidence indicates that an increased abundance of *Actinobacteria* is positively correlated with obesity (Wu et al., 2011) and adipogenesis (Wieland et al., 2015). An animal study demonstrated that the microbiota of high-fat diet (HFD)-fed mice is characterized by multiple substantially increased bacteria, including *Actinobacteria* (Ke et al., 2020). Another animal research also found that *Actinobacteria* is considerably more abundant in the HFD-fed group after 4 weeks of diet consumption and that a variety of pathogenic bacteria of such phyla could incite inflammation (Li et al., 2020). Similarly, our MR results are in agreement with a previous study that also reported that liver lipid content, including TC and LDL-C, was positively associated with *Actinobacteria*. Therefore, *Actinobacteria* may play an important role in promoting the development of hyperlipidemia. *Actinobacteria* are the most efficient biocatalysts of steroid transformation, such as deacetylation and bioconversion from steroid ketones to steroid alcohols (Donova, 2007), so increased serum TC levels might be due to increased intestinal *Actinobacteria* abundance.

The result of our MR analysis revealed that genus *Oscillospira* is considerably associated with lower TG and promisingly related to lower TC, suggesting a possible protective factor for host health. This finding is consistent with previous studies. *Oscillospira* is a common and beneficial bacterial genus that can be found throughout the animal and human intestines but has not been purely cultured. It has been reported that the *Oscillospira* abundance is strongly related to obesity, leanness, and human health (Sweeney and Morton, 2013). Besides, growing evidence indicates that *Oscillospira* is capable of producing all kinds of SCFAs dominated by butyrate, which can act as an important reference indicator to identify new probiotics (Yang et al., 2021). Chen et al. conducted multivariate association analysis using data from the Guangdong Gut Microbiome Project, in which they found that *Oscillospira* is positively associated with HDL-C and sleep time and negatively related to SBP, DBP, TG, fasting blood glucose, and uric acid (Ran et al., 2020). Their correlation results are highly consistent with the causal conclusions of our MR analysis. In addition, previous studies have also shown that *Oscillospira* plays an important role in the regulation of numerous metabolic processes associated with obesity and dyslipidemia (Ji et al., 2022). Thus, the results of our MR study further demonstrate the associations between *Oscillospira* and reduced serum lipid levels as well as improved dyslipidemia.

Our study has several advantages. First, the largest publicly available GWAS statistics of the gut microbiome were used in the two-sample MR analysis. Second, the causal estimates are reliable and stable owing to the strict quality control procedures and robust MR methods applied. Third, the potential causal associations identified by the IVW method may provide candidate bacterial taxa for future functional studies on the mechanism of the associations between gut microbiome with serum lipids.

Our study may also have some limitations. First, due to the design of the MR study, the data obtained from public databases were not individual-level statistics, which might lead to biases in causal estimates. Second, MR analysis strongly relies on its three assumptions of instruments, which are always difficult to completely satisfy despite the strict quality control conditions and sensitivity analysis. Third, while MR methods may provide novel insights into the causalities from the exposure traits to the outcome traits, the magnitude of the associations may not be estimated accurately, and thus, more research is needed to confirm the findings. Fourth, since this MR analysis mainly focused on populations with European ancestry, without extrapolating the results to other ethnic groups might also be a limitation of our study.

In conclusion, this MR study supports the positive and negative causal effects of gut microbiota on serum lipid levels. Our MR study showed that phylum Actinobacteria is causally related to LDL-C, genus *Terrisporobacter* is responsible for higher LDL-C and TC levels, and phylum Actinobacteria and genus *Oscillospira* are associated with lower TG levels. These findings strengthen our knowledge of the associations between gut microbiota with blood lipids, which may provide novel insights into the strategies for dyslipidemia prevention.

## Data availability statement

Publicly available datasets were analyzed in this study. This data can be found here: All data included in this MR analysis were obtained from publicly available datasets. The main summary statistics for gut microbiota can be downloaded from the MiBioGen database (https://mibiogen.gcc.rug.nl/menu/main/home/) and the human blood lipids datasets are publicly available in Global Lipids Genetics Consortium Results (http://csg.sph.umich.edu/willer/public/glgc-lipids2021/). The candidate datasets for gut microbiota can be obtained from the NHGRI-EBI GWAS Catalog (https://www.ebi.ac.uk/gwas/).

## Author contributions

GG designed the study, performed the data analysis, and drafted the manuscript. YW performed the data analysis and drafted the manuscript. YL and ZW performed the data analysis and revised the manuscript. GX performed the data analysis. XW and FL collected and cleaned the data. WL performed the replication analysis and helped revise the manuscript. QZ and XX assisted in carrying out the study and critically revised the manuscript. SZ designed the study, led the study, and revised the manuscript. All authors reviewed and approved the final manuscript.

## Funding

This study was funded by the National Natural Science Foundation of China (Nos. 82274016, 81872934, 81903652, and 81673514), the Science and Technology Planning Project of Guangdong Province, China (No. 2017B030314041), Science and Technology Development Projects of Guangzhou (No. 202201011424), the Guangdong Provincial Key Laboratory of Coronary Heart Disease Prevention (No. Y0120220151), and Science and Technology Development Projects of Guangzhou, Guangdong, China (No. 202002030415).

## Conflict of interest

The authors declare that the research was conducted in the absence of any commercial or financial relationships that could be construed as a potential conflict of interest.

## Publisher’s note

All claims expressed in this article are solely those of the authors and do not necessarily represent those of their affiliated organizations, or those of the publisher, the editors and the reviewers. Any product that may be evaluated in this article, or claim that may be made by its manufacturer, is not guaranteed or endorsed by the publisher.
